# Comparing the effectiveness of 3D printing technology in the treatment of clavicular fracture between surgeons with different experiences

**DOI:** 10.1186/s12891-022-05972-9

**Published:** 2022-11-22

**Authors:** Meng Zhang, Jianglong Guo, Hongyi Li, Jingzhi Ye, Jun Chen, Jingfeng Liu, Mengqiang Xiao

**Affiliations:** Zhuhai Hospital, GuangdongProvincial Hospital of Traditional Chinese Medicine, 53 Jingle Road, Zhuhai City, Guangdong Province China

**Keywords:** 3D Printing, Clavicular, Fracture, Different experiences

## Abstract

**Purpose:**

This study aims to examine the use of 3D printing technology to treat clavicular fractures by skilled and inexperienced surgeons.

**Methods:**

A total of 80 patients with clavicle fractures (from February 2017 to May 2021) were enrolled in this study. Patients were divided randomly into four groups: group A: Patients underwent low-dose CT scans, and 3D models were printed before inexperienced surgeons performed surgeries; group B: Standard-dose CT were taken, and 3D models were printed before experienced surgeons performed surgeries; group C and D: Standard-dose CT scans were taken in both groups, and the operations were performed differently by inexperienced (group C) and experienced (group D) surgeons. This study documented the operation time, blood loss, incision length, and the number of intraoperative fluoroscopies.

**Results:**

No statistically significant differences were found in age, gender, fracture site, and fracture type (*P* value: 0.23–0.88). Group A showed shorter incision length and fewer intraoperative fluoroscopy times than groups C and D (*P* < 0.05). There were no significant differences in blood loss volume, incision length, and intraoperative fluoroscopy times between group A and group B (*P* value range: 0.11–0.28). The operation time of group A was no longer than those of groups C and D (*P* value range: 0.11 and 0.24).

**Conclusion:**

The surgical effectiveness of inexperienced surgeons who applied 3D printing technology before clavicular fracture operation was better than those of inexperienced and experienced surgeons who did not use preoperative 3D printing technology.

## Introduction

A clavicle fracture is one of the common clinical diseases. Operative plate fixation is the primary method for clavicle fracture treatment [1, 2]. The clavicle is a slender S-shaped long bone with a superficial position. Its morphology varies significantly among individuals, leading to poor suitability of standard internal fixation. Therefore, using a 3D printed model to assist internal fixation selection and surgical plan formulation seems essential [3] in this field.

With the rise of 3D printing technology in recent years, orthopedic surgeons have produced several surgery plans based on conventional CT pictures and 3D-printed models. In addition, surgeons have recently begun to apply 3D printing technology in almost all areas of orthopedic trauma surgery [4, 5]. By creating specific 3D printed anatomical models, surgeons can better understand the anatomical structure details of patients' fractures, including trauma bone, surrounding soft tissue, and normal areas. Thus 3D printing technology is helping to make accurate preoperative plans [6–10].

The global unmet need for surgery, estimated to be over 320 million surgical procedures per year, is concentrated primarily in low- and middle-income countries due to a lack of medical resources [11, 12]. Less than half of the minimum needed procedures were done in low- and middle-income countries. One main reason is that surgeons have limited surgical skills [11–14].

In this study, we simulated the surgical effectiveness of preoperative 3D printing by inexperienced surgeons in low- and middle-income countries, and the results were compared with those of surgeons with different experiences who only used preoperative CT evaluation.

## Materials and methods

### Study population

**Fig. 1:**
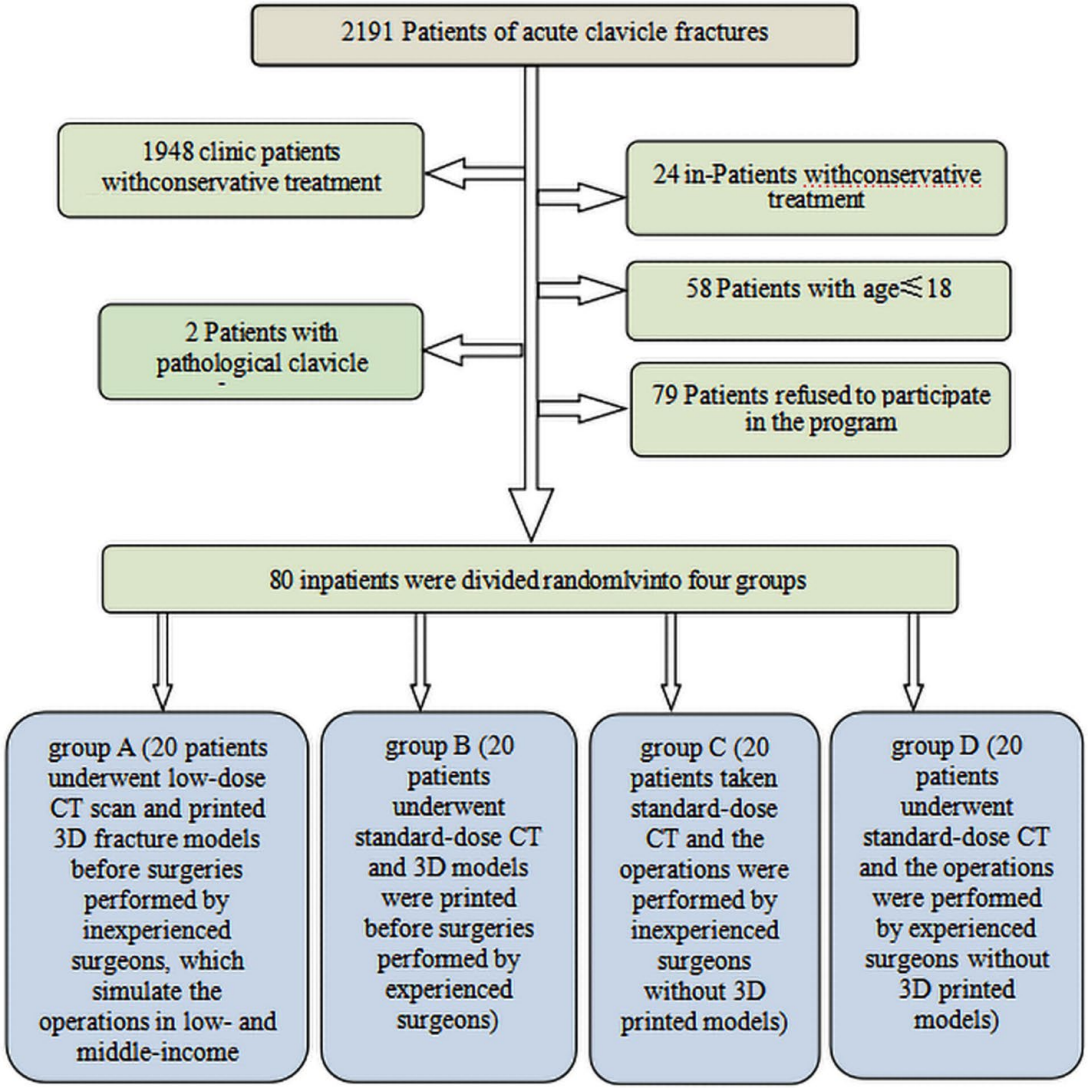
Flowchart of the study

This study was approved by the institutional review board of the Guangdong Hospital of Traditional Chinese Medicine (BF2019-030–01). All patients with clavicle trauma admitted for preoperative imaging examination were included in this study (from February 2017 to May 2021). Inclusion criteria were as follows: age ≥ 18 years, signed written consent, and patients with a suspected fractured shoulder from the emergency department. Exclusion criteria included: age < 18 years, patients with conservative treatment, patients with pathological clavicle fractures, and patients who rejected to provide written informed consent to participate in the study (Fig. 1). The study consisted of 80 patients (mean age 43.02 years; range 18–78 years), including 60 men and 20 women (Table 1).

**Table 1:** Patients' demographic and baseline characteristics

group	A	B	D	C	P
No	20	20	20	20	
Age	45.50 ± 13.41	37.40 ± 15.68	44.1 ± 10.75	44.55 ± 13.65	0.42
Gender	14 M 6F	17 M 3F	17 M 3F	12 M 8F	0.23
Fracture site (middle /Distal, case)	15/5	17/3	18/2	18/2	0.65
Comminuted fracture(case)	17	15	17	17	0.88

A total of 80 inpatients were divided randomly into four groups (Fig. 1): group A (20 patients underwent low-dose CT scan and 3D-printed fracture models before surgeries performed by inexperienced surgeons, simulating the operations in low- and middle-income countries), group B (20 patients underwent standard-dose CT, and 3D models were printed before surgeries performed by experienced surgeons), group C (20 patients took standard-dose CT and the operations were performed by inexperienced surgeons without 3D printed models), and group D (20 patients underwent standard-dose CT and the operations were performed by experienced surgeons without 3D printed models). The surgeons in groups A and C and those in groups B and D were identical. The operation time, blood loss (weighed gauze [15]), incision length, and the number of intraoperative fluoroscopies were recorded in four groups.

### Image acquisition and production of 3D printed model

Patients with clavicular fracture were scanned by Toshiba 640-slice CT (Aquilion One; Canon Medical Systems, Otawara, Japan) and were randomly assigned to groups A, B, C, and D (Fig. 1). In group A, the image quality simulated the low-grade CT in a primary hospital (low-grade Philips MX16-Slice CT in the Second People's Hospital of Xiangzhou District; tube voltage 120 kV, tube current 280mAs). Images of 36 patients with clavicular fractures in the Second People's Hospital of Xiangzhou District were low-grade CT images selected in group A. The CT image quality was scored using a five-point Likert scale: 1 = extremely poor; 2 = poor; 3 = fair; 4 = good, and 5 = excellent. Images ≥ 3 points were regarded as acceptable quality. No significant difference in image quality score was found between group A and group in the Second People's Hospital of Xiangzhou District (4.40 ± 0.60 vs. 4.36 ± 0.55, *p* = 0.59). The CT scanning tube voltage is 120 kV, and the tube current is 150 mAs in Groups B, C, and D (Table 2).

**Table 2:** CT parameters of each group

Dose group	Standard	Ultra-low	P
Tube voltage (kV)	120	100	
Tube current (mA)	150	140	
D-FOV(mm)	500	500	
Rotation time (s)	1.00	0.75	
Thickness (mm)	0.50	0.50	
Interval (mm)	0.50	0.50	
Scan length (cm)	160.00	160.00	
AIRD3D	Standard EU10	Standard EU10	
CTDIvol(mGy)	14.57 ± 0.60	6.67 ± 0.25	0.01
ED(mSv)	2.17 ± 0.36	1.10 ± 0.13	0.01

Preoperative CT images with 0.5-mm slice thickness were imported to Mimics 22.0 software for reconstruction. The geometrical bones from the CT images were segmented using Mimics 22.0 software (Materialise, Leuven, Belgium). The segmented file was then transferred to the 3-Matic 15.0, a modulus in Mimics 22.0, to perform the virtual fracture reduction to normalized anatomy. These 3D models (saved as MCS format) were further transferred in Gcode format using specific software provided by the manufacturer of the 3D printer (Tianwei ColiDo 3.0, China). The printing material was polylactic acid. The resolution of the printed models was 0.011 mm.

Groups A and B used the PLA materials for 3D fracture model printing. Digital models were generated from 3D-reconstructed CT data at 0.5-mm slice intervals using MICs Research software, version 22.0. The skeleton model was printed by the melting deposition method on a CoLiDo 3.0 3D printer using white polylactic acid with a diameter of 1.75 mm.

### 3D printed models evaluation

The double-blind five-point Likert scale was used to analyze the 3D printed models' quality. 3D models with good quality and smooth surfaces scored excellent (5 points). Models with slightly blurred surfaces were scored as good (4 points). Models with rough surfaces but providing sufficient information for the preoperative decisions were scored as far (3 points). When the surface quality was too coarse to provide enough information, it was scored as poor (2 points). Finally, the model was scored as extremely poor (1 point) when it could barely provide useful information.

Operators with more than 15 years of experience were considered experienced, while those with experience of fewer than five years were regarded as inexperienced. When the two surgeons had different opinions, adding another surgeon with more than ten years of orthopedic surgery experience was involved in making the final decision.

One experienced surgeon and one inexperienced surgeon were randomly assigned to assess image quality in each patient. When the two surgeons had different opinions, they were to negotiate and reach a consensus.

### Statistical analysis

Statistical analysis was performed using SPSS (IBM Corp, Armonk, NY, USA, version 26.0). One-way ANOVA and independent-sample T-test were used to determine if there were differences in patient age, operation time, blood loss, incision length, number of intraoperative fluoroscopies, CT image quality score, and 3D printing model score. In addition, the two surgeons used the ICC test to analyze the consistency of the 3D model and the image quality evaluation. The ICC values were defined as follows: < 0.4 slightly consistent; 0.41 to 0.60, moderately consistent; 0.61 to 0.80, highly consistent; and 0.81 to 1.00, almost perfectly consistent. A difference with a *p*-value < 0.05 indicates statistical significance.

## Results

No statistically significant differences were found in age, gender, fracture site, and fracture type (Table 1, *P* value: 0.23–0.88). The CT image quality score of group A was 4.40 ± 0.60, which was less than that of the other three groups (*P* < 0.05). The CT image quality of one case in group A was 3 points, and the rest were ≥ 4 points, indicating good image quality. All cases of the other three groups were 5 points, indicating excellent image quality, which could meet the clinical needs. Only one case of the 3D printing model in group A was evaluated as 4 points, while the other 3D printing models (in four groups) were evaluated as 5 points. ICC was 0.89 in group A for CT image quality and 3D printing model evaluation. ICC was 0.99 for CT image quality evaluation and 3D printing model evaluation in groups B, C, and D.

The operation time, blood loss, incision length, and the number of intraoperative fluoroscopy of each group are shown in Table 3. Group A showed shorter incision length and fewer intraoperative fluoroscopy times than groups C and D (*P* value range: 0.01–0.01). There were no significant differences in blood loss volume, incision length, and the number of intraoperative fluoroscopy times between group A and group B (*P* value range: 0.11–0.28). The operative time of group A was no longer than that of groups C and D (*P* values: 0.11 and 0.24).

**Table 3:** Surgical effect and image quality were evaluated in each group

Dose group	A	B	C	D	P
operation time (min)	103.65 ± 26.94	83.60 ± 19.30	117.50 ± 26.75	102.50 ± 25.31	0.01
blood loss (ml)	24.00 ± 14.74	17.25 ± 10.82	31.50 ± 21.34	27.00 ± 10.81	0.01
Length of incision(cm)	6.36 ± 2.37	7.25 ± 1.97	9.80 ± 1.61	9.15 ± 1.84	0.01
number of Intraoperative fluoroscopy (n)	5.95 ± 1.05	5.25 ± 0.97	13.85 ± 3.05	9.25 ± 2.27	0.01
CT image quality assessment (fraction)	4.40 ± 0.60	5.00 ± 0.01	5.00 ± 0.01	5.00 ± 0.01	0.01
3D fracture model(fraction)	4.95 ± 0.224	5.00 ± 0.01	5.00 ± 0.01	5.00 ± 0.01	0.30

## Discussion

This experiment simulated the preoperative image acquisition and surgical treatment of clavicle fracture in low- and middle-income countries. No difference was found in our pre-experiment comparing the image quality of high-grade low-dose CT with that of low-grade CT. In this study, low-dose CT scanning was performed in our hospital to simulate low-grade CT image quality in economically underdeveloped areas, and 3D models were printed. To simulate the surgeons with limited surgical skills in low- and middle-income countries, inexperienced surgeons in our hospital made surgical plans (group A) based on low-dose CT images and 3D printing models. Meanwhile, the operation time, blood loss, incision length, and the number of intraoperative fluoroscopies were recorded. No significant difference was found in operation time and blood loss between inexperienced doctors who used low-dose CT and 3D printing models (group A) and experienced surgeons who used conventional CT (group D). However, the former group exhibited significantly shorter incision length and lesser intraoperative fluoroscopy time than the latter group. Based on the conventional CT images, the surgical effect of experienced surgeons was better than inexperienced surgeons.

3D printing technology assisted in treating internal fracture fixation and showed an excellent surgical effect, confirmed in many studies [7–10, 16, 17]. In the past, the large size and high price of 3D printing machines and materials [17] made it difficult for 3D printing technology to be popularized in hospitals in low- and middle-income countries. Recently, 3D printing technology has been widely used in fracture surgery due to lower manufacturing costs, fewer printing steps, and lesser complexity, providing conditions for hospitals in low- and middle-income countries to use this technology. A standard home edition 3D printing machine was used in this study. The printing accuracy was 0.06–0.50 mm, which could meet the needs of orthopedic surgery (Double Medical Internal fixation size 83.00–160.00 mm, the size difference between adjacent models of the former was 4.00–12.00 mm). The relatively low-cost material PLA (Tianwei Co., Ltd., US $9.83 for 1 KG PLA material) was used in this study. Printing one 3D clavicle model costs about $0.84 and takes about 3.01 ± 0.20 h. Shuang F et al. printed one elbow joint model for about $3.00, which took about four hours [16]. In our study, 3D printing process self-study videos were provided by 3D printing manufacturers, and 3D modeling parameters were adjusted remotely by the manufacturers. Ordinary computers could run the software, which was easy to learn.

**Fig. 2:**
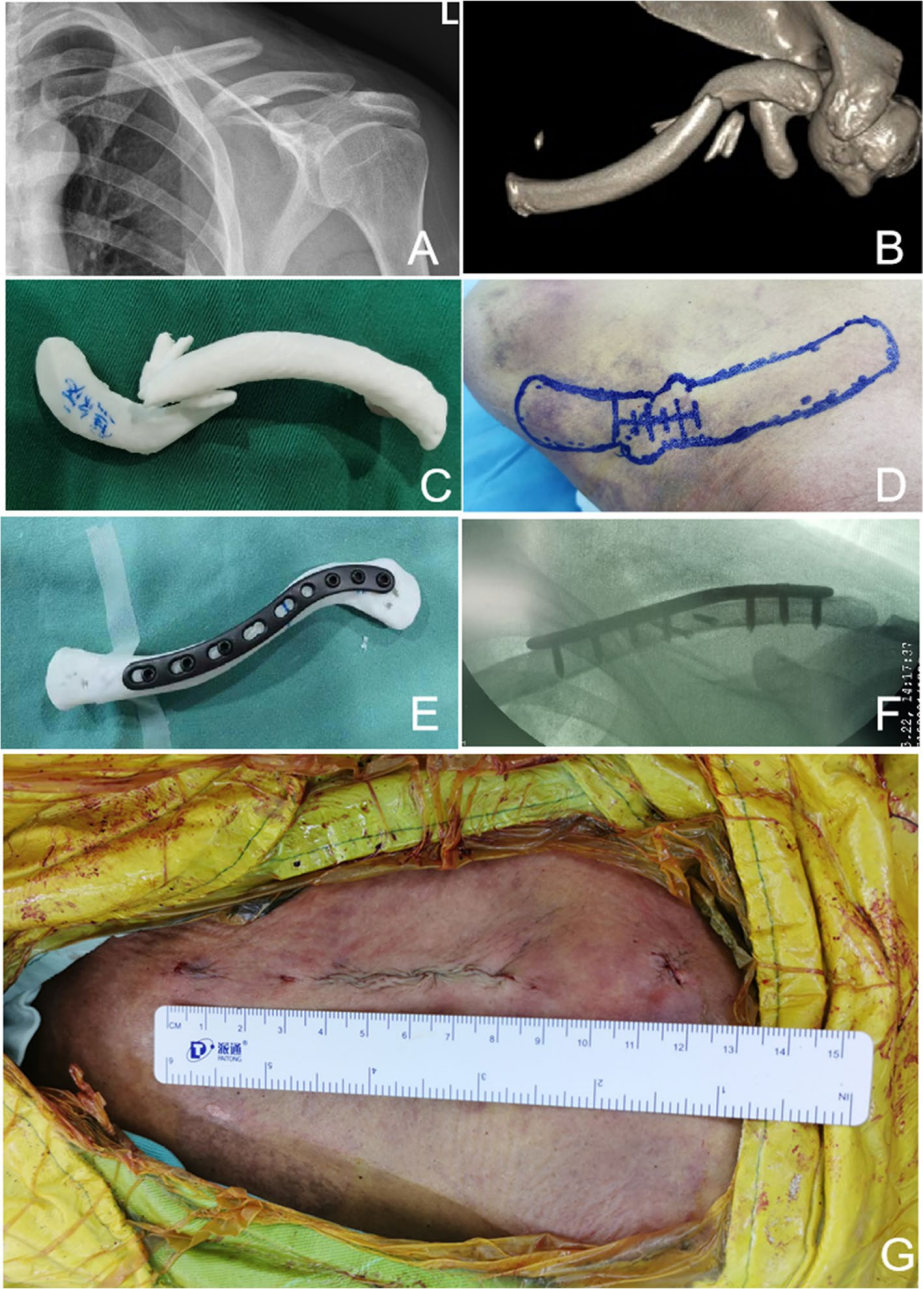
A 53 years old male with middle site clavicle fracture. The operation time was about 100 min, and the incision length was 8.30 cm. The blood loss was 10 mm during the operation, and intraoperative fluoroscopy was performed five times. (2a) DR image; (2b) CT 3D-reconstruction; (2c) 3D printing model of clavicle fracture; (2d) preoperative marking; (2e) The internal fixation selection and preoperative delineation of clavicle fracture site; (2f) Intraoperative fluoroscopy of clavicle fracture; (2 g) incision length (8.30 cm)

In the current study, many scholars studied the application of 3D printing technology to assist fracture surgery and achieved sound surgical effects. However, none of them studied in groups according to surgeons' experiences [5, 8, 16, 18, 19]. Our study compared the operation effectiveness of inexperienced and experienced surgeons using 3D printing technology and routine CT images before operations with the same inexperienced and experienced surgeons only using routine CT scans before operations. The former has less incision length and intraoperative exposure. Since 3D printing models could provide a visual, comprehensive vision of fracture, the position of plate implantation, screw direction, and screw length can be determined in the simulation operation before operation. Therefore, when Group A simulated low-grade CT scan conditions in underdeveloped areas and with the assistance of a 3D printing model, the operation time and blood loss of inexperienced surgeons were no different from those of inexperienced and experienced surgeons with conventional CT-assisted operations. 3D printing could supplement routine CT scans, allowing surgeons to understand patients' fractures more intuitively and achieve better surgical results (Fig. 2).

The limitations of this study are as follows: 1. This study only simulated the environment where hospitals equipped with low-grade CT and surgeons were inexperienced in economically underdeveloped areas and did not collect patients in economically underdeveloped areas. 2. The internal fixation in this study is not a customized internal fixation but a relatively appropriate internal fixation selected from a series of sizes. 3. Due to the sample size of our study, we did not divide patients into subgroups using AO/OTA-2018 classification. 4. A comparison between the 3D-printed model and the CT scan might be added to evaluate the accuracy of the model's dimensions in relation to the CT scan's dimensions.

## Conclusion

The surgical efficacy of inexperienced surgeons who applied 3D printing technology before clavicular fracture operation was better than that of inexperienced and experienced surgeons who did not use preoperative 3D printing technology. The use of 3D models was beneficial for inexperienced surgeons making preoperative plans.

## Data Availability

The datasets used or analyzed during the current study are available from the corresponding author upon reasonable request.
